# Impact of green space activity on older adults’ mental health in “double-aging” communities: a case study of Shangcheng District, Hangzhou

**DOI:** 10.3389/fpubh.2026.1871001

**Published:** 2026-06-09

**Authors:** Jiao Zhang, Yichen Jiang, Danqing Li

**Affiliations:** 1 School of Fine Arts, Hangzhou Normal University, Hangzhou, Zhejiang, China; 2 Interdisciplinary Research Center for Digital Intelligence Design, School of Fine Arts, Hangzhou Normal University, Hangzhou, Zhejiang, China; 3 School of Horticulture, Chiba University, Matsudo, Chiba, Japan; 4 School of Civil Engineering and Architecture, Zhejiang Sci-Tech University, Hangzhou, Zhejiang, China

**Keywords:** double-aging communities, green space activity, green space justice, mental health, structural equation modeling

## Abstract

With the rapid aging of China’s population, so-called “Double-Aging” communities—characterized by a high proportion of older residents and relatively aged buildings—are becoming increasingly prevalent in urban areas. This study takes Shangcheng District in Hangzhou as a case, systematically examining the effects of community green space, green space activity behaviors, and community renovation on older adults’ mental health across different community types, with particular focus on double-aging communities. Communities were pre-selected based on publicly available government and spatial data, followed by a questionnaire survey yielding 312 valid responses, covering individual characteristics, green space features, activity behaviors, and mental health indicators. Data were analyzed using structural equation modeling (SEM), hierarchical regression, ANOVA, and independent-sample *t*-tests. Results indicate that green space planning indirectly promotes older adults’ mental health by enhancing overall perception, social interaction, and activity engagement, while social interaction frequency and activity participation have significant direct positive effects. Community renovation significantly improves overall perception and mental health, with pronounced benefits for middle-aged older adults (55–65 years), but limited effects for the oldest group (65 years and above). Moreover, heat-related environmental factors partially attenuate these positive effects. Overall, the findings suggest that optimizing green space planning, improving public environments, promoting social interaction and activity participation, and incorporating heat-adaptive strategies can effectively enhance older adults’ mental health. The study provides theoretical and practical implications for the planning and policy-making of double-aging communities and highlights the need for future research to consider high-aged residents’ adaptability, longitudinal analyses, and the integration of multidimensional environmental factors.

## 1 Introduction

### 1.1 The “double-aging” phenomenon in Chinese cities

As China’s population continues to age rapidly, the “double-aging” phenomenon in urban communities has become increasingly prominent (1–3). The term “double-aging” typically refers to a dual condition at the community level, where demographic aging and aging of the built environment overlap. On one hand, the proportion of older adults continues to rise; on the other hand, community buildings and supporting facilities enter a phase of physical deterioration (1, 4, 5).

According to projections by the National Bureau of Statistics of China, by 2030 the proportion of the population aged 65 and above is expected to increase significantly, further deepening the degree of aging (6). Generally, a region is considered to have entered a stage of “moderate aging” when the proportion of residents aged 65 and above exceeds 14% (7, 8). By the end of 2024, the national proportion of people aged 65 and above had reached 15.6%, marking the official entry into a deeply aging society (2, 9). At the urban level, the Northeast exhibits the highest aging, followed by Sichuan-Chongqing, while the Yangtze River Delta is rapidly aging, especially in core cities (10–12). The proportion of registered residents aged 60 and above in Shanghai reaches as high as 37.6% (13), while Hangzhou, and Suzhou have resident older adult populations exceeding 14%, crossing the threshold for deep aging (14, 15). In cities such as Shanghai and Hangzhou, the inflow of younger labor partially offsets the pressure of aging among permanent residents, resulting in registered population aging rates that are significantly higher than those of the resident population (14, 68). However, this “siphoning effect” implies that surrounding cities may experience accelerated outflow of young residents, thereby speeding up the aging process in those areas.

Meanwhile, the rapid urbanization process has left a large number of aging residential communities, further amplifying the “double-aging” challenge arising from the overlap of demographic and built environment aging. Most of these communities were constructed during the planned economy period or the early phase of market reforms, consisting primarily of work-unit dormitories or early commercial housing. Their spatial layouts and facility configurations are inherently constrained by the economic conditions, planning concepts, and construction standards of their time. As the buildings age, old communities generally face structural deterioration, degradation of municipal infrastructure, and insufficient provision of basic public services. Additionally, they commonly exhibit compact spatial layouts, limited functional diversity, inadequate circulation and activity spaces, and a lack of accessible and age-friendly infrastructure. The insufficiency of basic elder-adaptation measures is particularly pronounced (16, 17).

At the level of green public spaces, most aging communities nationwide exhibit insufficient green area, low landscape quality, and uneven spatial distribution. Provision of micro-scale recreational green spaces and pedestrian activity areas is particularly inadequate (18, 19). Survey data indicate that older adults primarily conduct daily activities within a 15- min walking radius of their residences, relying heavily on nearby outdoor leisure and wellness spaces (20, 21, 67). However, the limited quantity and quality of green spaces in aging communities fail to meet the routine health, leisure, and social needs of older residents. Furthermore, these communities are often located in central or well-developed urban areas, characterized by high population density, scarce land resources, and limited space for redevelopment. Compounded by complex property rights, difficulties in raising renovation funds, strict regulatory constraints, and high coordination costs among stakeholders, the systemic renewal and age-friendly adaptation of old communities face significant implementation barriers (21, 22).

### 1.2 Urban stock renewal and green space justice

Under the dual constraints of an aging population and deteriorating material environments, “double-aging” communities have become a typical spatial type in the stock renewal phase of large Chinese cities. As China’s urban development model shifts from an “expansive” to a “consolidative” approach, the focus of urban spatial governance is concurrently moving from the expansion of incremental space to the enhancement of existing stock (23, 24). The renewal of aging communities, as a core component of stock space governance, has therefore become increasingly important (25).

Since the launch of the national urban renewal program in 2019, nearly 280,000 old urban residential communities have undergone renovation, benefiting approximately 120 million residents and including the installation of over 130,000 elevators. By 2024, renovation efforts continue, with plans to initiate approximately 54,000 projects, while local reports indicate that about 58,000 communities will be newly renovated nationwide within the year. According to Ministry of Housing and Urban–Rural Development of China, all urban residential complexes constructed before 2000 are to be included in the renovation scope, with implementation adapted to local conditions (25, 26). Local authorities are also encouraged to explore resident-led initiatives for upgrading old residential buildings. As the type of old residential community characterized by the most prominent aging population and the most severe environmental deterioration, “double-aging” communities represent both a priority and a challenge in urban renewal initiatives.

“Green space justice,” as a concrete manifestation of urban justice in the allocation of green areas, primarily concerns ensuring that different groups—particularly vulnerable users such as the older population—have equal rights to use, access, and benefit from community green spaces. It emphasizes the fairness of green space allocation, the rationality of spatial layout, and the adaptability of functional provision (27–29, 69). In “double-aging” communities, issues such as insufficient green space, uneven quality, and significant differences in accessibility essentially reflect a lack of green space justice, which further exacerbates the constraints that the “double-aging” problem imposes on the quality of life of older residents (30).

### 1.3 Green space activities and older adults mental health

Research indicates that improving the accessibility and usability of community green spaces not only enhances environmental quality but also exerts positive effects on psychological wellbeing, social engagement, and daily life convenience, thereby strengthening overall community resilience and livability (31, 32, 70). In the context of stock space renovation, fully leveraging the catalytic and ecological effects of community green spaces is a critical approach to improving the quality of renewal projects and adapting to the needs of older residents (33, 34).

Empirical studies have confirmed that green environments significantly promote residents’ mental health. Particularly, small-scale green spaces such as community gardens and street-corner parks can maximize the use of limited space, compensate for the “inequity of green space” present during early urban planning stages, and provide convenient locations for daily rest and social interaction (35, 36). Regular exposure to green environments can alleviate psychological stress, reduce depressive symptoms, enhance positive emotional experiences, and to some extent stimulate physical activity and social interaction, thereby fostering harmonious neighborhood relationships (35, 37, 40). For example, during winter or cold climates, sunlight exposure and vegetation in green spaces can effectively mitigate feelings of loneliness and depression, improving mental wellbeing (38). Conversely, during hot summer periods, the lack of comfortable outdoor green spaces may reduce residents’ outdoor activities, exacerbating “air-conditioning sickness,” while high-temperature outdoor activity may increase the risk of heat-related illnesses, posing physical and mental health threats (39).

For the older adults, natural declines in physical function and limited mobility result in a relatively fixed activity range and smaller spatial radius, accompanied by fewer social opportunities. Consequently, their risk of mental health issues is significantly higher than that of other age groups (40, 41). In modern information-based societies, traditional extended-family arrangements based on agricultural production and concentrated worker housing in industrial societies have gradually disintegrated, sharply reducing the frequency of daily contact between older adults and their children or relatives (42, 43). This social structural change further exacerbates loneliness and increases the likelihood of mental health problems (35, 44). In “double-aging” communities, the aging residential environment, insufficient provision of public green space, and irrational spatial layout—compounded by unequal resource distribution stemming from the lack of green space justice—amplify the spatial vulnerability of older residents, creating new potential health risks and undermining their mental wellbeing.

As an integral component of urban stock space, the renewal of “double-aging” communities—characterized by both a high proportion of older residents and aging physical infrastructure—involves a wide range of urban contexts in China. However, while health-promotion-oriented spatial planning emphasizes the rational layout of public green spaces to meet the needs of aging populations and achieve environmental equity, existing literature has rarely addressed the mental health of older adults within this specific “double-aging” context. The specific mechanisms through which green space activities influence mental health in these environments remain underexplored. To address this research gap, this study investigates the relationship between green space activity behaviors and older adults in these communities. The specific contributions of this study are threefold: (1) examining the effects of green space activities on older adults’ mental health specifically within the context of “double-aging” communities; (2) applying structural equation modeling (SEM) to investigate the complex mechanisms linking community spatial layout, green space use, and mental health outcomes; and (3) providing evidence-based implications for community planning and public health interventions. By identifying the core issues linking spatial design to residents’ health, this study provides practical evidence for optimizing health-promotion-oriented renovation, improving residents’ quality of life, and achieving healthy city development objectives (45).

## 2 Methods

### 2.1 Study area

**Figure 1:**
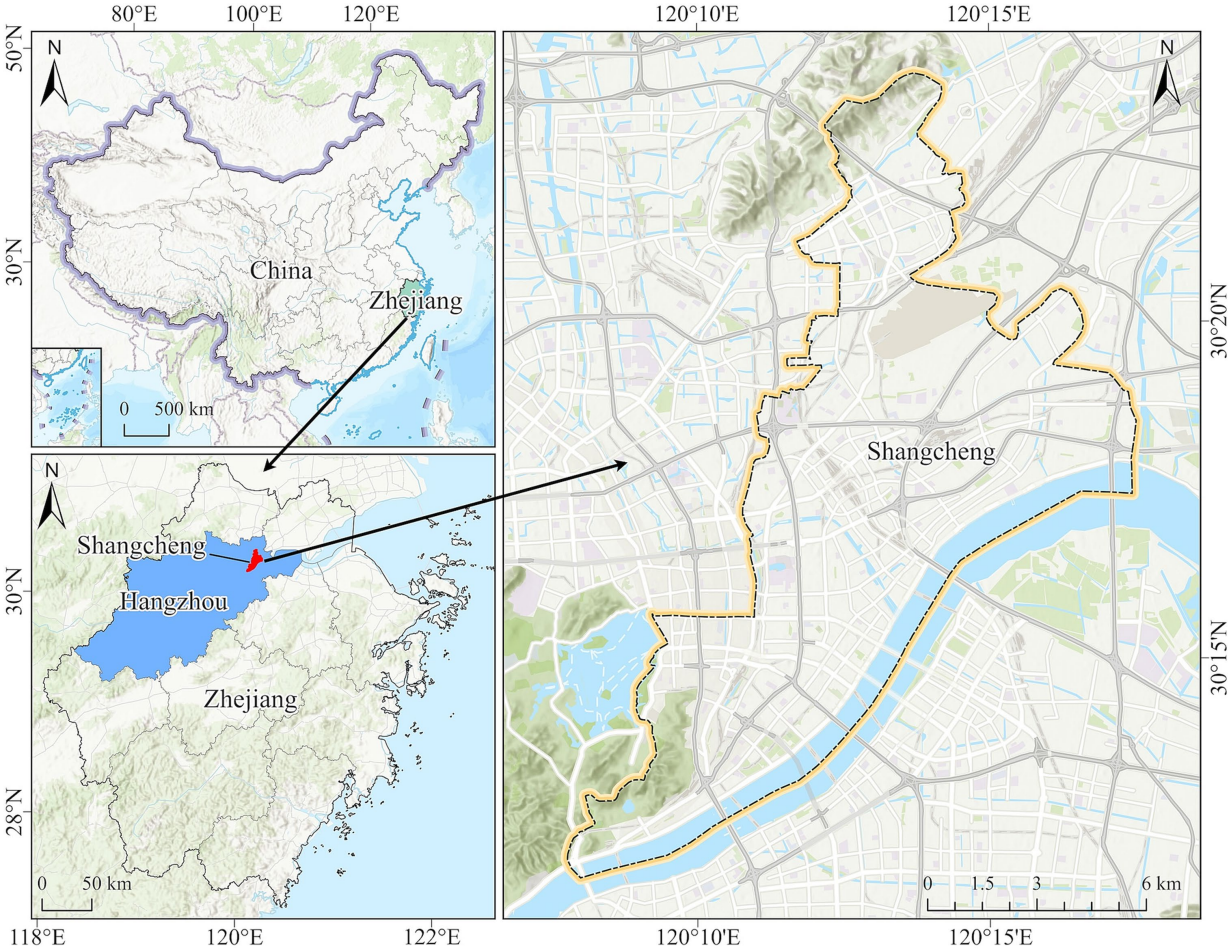
Location of the study area.

This study selects Hangzhou as the research area and Shangcheng District as the specific site for empirical investigation. As the capital of Zhejiang Province and a core city within the Yangtze River Delta urban agglomeration, Hangzhou exhibits typical characteristics of population aging in major eastern Chinese cities (Figure 1). Moreover, the distribution of “double-aging” communities is concentrated, and the inequitable allocation of green space resources is pronounced, making it a suitable area for this study. According to the 2024 Key Population Data Bulletin released by the Hangzhou Municipal Government, by the end of 2024, the city’s permanent population reached 12.624 million, of which 2.582 million were aged 60 and above, accounting for 20.5% of the total population. The population aged 65 and above was 1.772 million, representing 14.0% of the total. Compared with the end of 2023, the number of residents aged 60 and above increased by 123,000, indicating a notable acceleration in the aging process (14).

Shangcheng District, as one of Hangzhou’s earliest developed core districts, has a full urbanization rate of 100%. Covering a total area of 122 km^2^ and administratively divided into 14 subdistricts, the district had a permanent population of 1.398 million at the end of 2024, with a population density of approximately 11,459 persons per km^2^. Among them, around 258,300 residents were aged 60 and above, accounting for 27.9% of the total population, a level of aging significantly higher than the citywide average (46). The building stock in Shangcheng District is generally aged, making it one of the districts in Zhejiang Province with the largest number of old residential communities and the highest renovation difficulty. Even residential buildings constructed around 2000 face noticeable shortcomings in living quality due to the limited construction budgets and low property management standards of that era.

In this context, Shangcheng District exemplifies a “double-aging community” (double-aging communities), defined as neighborhoods where both population and residential buildings are relatively old. Population aging is operationalized as the proportion of residents aged 60 years and above, with a threshold of 14% based on China’s official definition of aging (7). Building aging follows the criteria of the Guiding Opinions on Comprehensively Promoting the Renovation of Old Urban Residential Communities, which highlights residential complexes built before 2000 (e.g., state-owned enterprise, government, or school dormitories), housing reform units (public housing sold before 1998), and early commercial housing developed before 2000 with relatively lower construction standards (47).

As one of Hangzhou’s earliest developed core districts, Shangcheng District contains a large number of buildings constructed before 2000, alongside numerous high-end residential complexes developed more recently. This combination has created a complex spatial pattern where old communities coexist with upscale housing, leading to notable disparities in facilities, living conditions, and green space availability. In this context, green space resources are unevenly distributed, reflecting a lack of green space justice and highlighting inequitable access to community resources in “double-aging” neighborhoods. Accordingly, Shangcheng District meets the criteria of a double-aging community, providing a policy-relevant and empirically typical setting for examining the relationship between green space availability and older adults’ mental health.

### 2.2 Community selection and questionnaire survey

In the community selection phase, this study employed multi-source spatial data for preliminary screening. The spatial distribution of the older population in Hangzhou was obtained from WorldPop (48), which enabled the initial identification of areas with high concentrations of older residents. Data on community greening rates were sourced from Copernicus Data Space to assess the level of green space coverage in each community (49). Information on building age was obtained through the Google Earth Engine platform, integrating historical imagery and building datasets to determine the construction period of residential communities (50).

**Figure 2:**
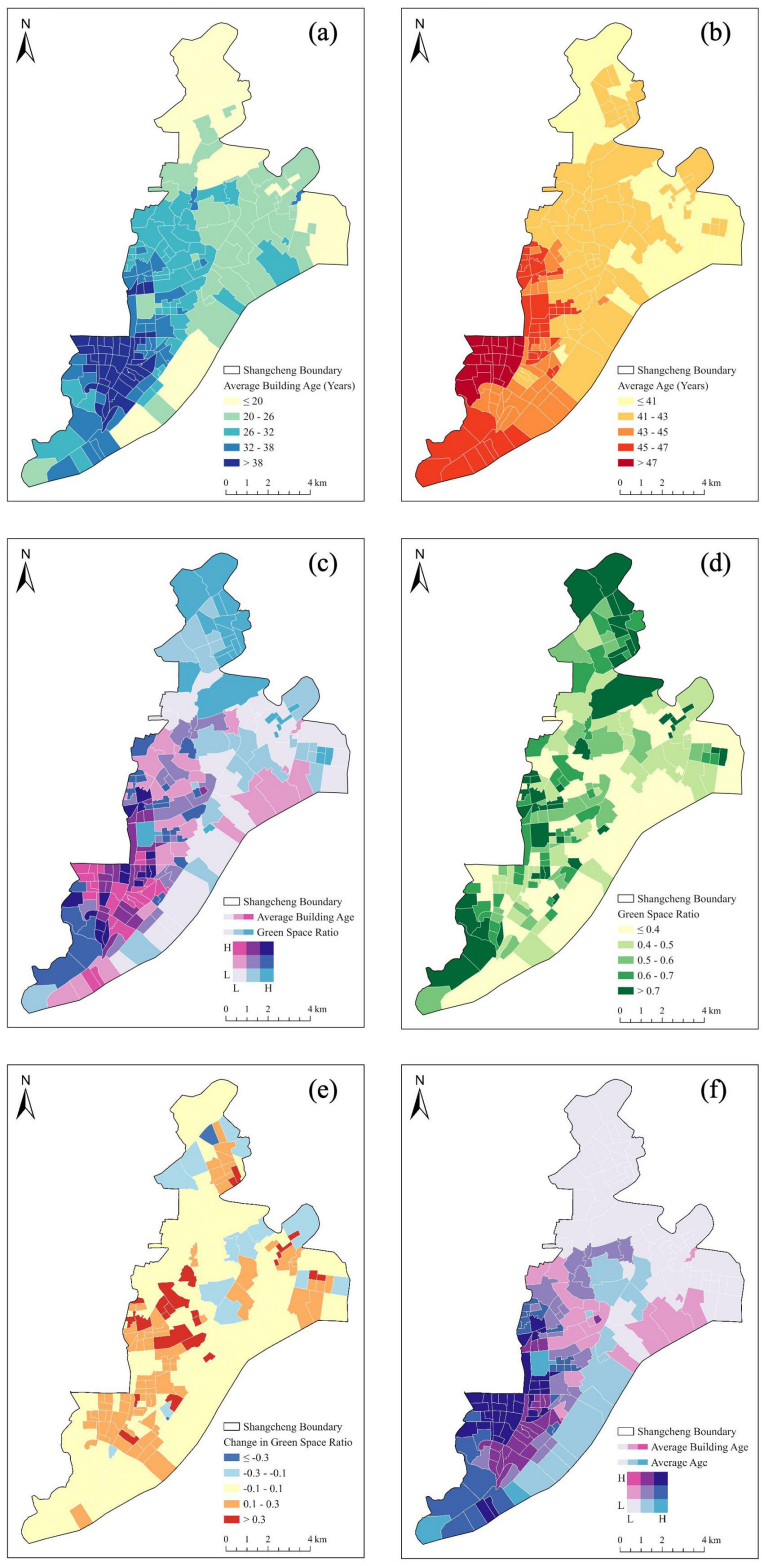
Spatial characteristics and classification of communities in Shangcheng District, Hangzhou: **(a)** building age distribution; **(b)** older population density; **(c)** community classification into LL, LH, HL, and HH types; **(d)** community green space coverage; **(e)** relationship between building age and green space ratio; **(f)** recent changes in green space coverage.

To facilitate community classification, preliminary GIS-based analyses were conducted to identify spatial patterns of building age (Figure 2a) and older population density (Figure 2b) across the study area. To capture the natural variation in these datasets, the natural break (Jenks) method was applied to determine thresholds for “high” and “low” categories in both dimensions. Based on these natural breaks, communities were stratified along two dimensions—building age and resident age structure—resulting in four types: low building age–low older proportion (LL), low building age–high older proportion (LH), high building age–low older proportion (HL), and high building age–high older proportion (HH) (Figure 2c). During the selection of specific communities for field surveys, care was taken to ensure that all four community types were represented, capturing the full spatial and structural variation in building age and older population density.

Data were collected from April 2024 to December 2025 across four distinct community categories, strategically selected to ensure that the environmental variables adequately reflected community heterogeneity. The survey was executed by four research subgroups, each comprising an average of 18 members. To meet the sample size requirements for SEM—which typically necessitates 5–10 times the number of measurement items—a target of 50–100 valid questionnaires per community type was established. Considering the limited mobility of some older residents, data were primarily gathered via face-to-face interviews and with assistance from family members to improve response rates and ensure data completeness. On average, each team member administered 4–5 questionnaires. After excluding incomplete or invalid responses, exactly 78 valid questionnaires were retained per community type, culminating in a final robust dataset of 312 valid questionnaires across all four categories.

Community green space coverage varied noticeably across different community types (Figure 2d). When combined with building age, older communities tended to have lower green space ratios (Figure 2e). By examining recent changes in green space, it is evident that some older communities have undergone renovation in recent years, resulting in significant improvements in their green space coverage (Figure 2f). Based on these observations, care was taken during the selection of specific communities to ensure that all community types were represented.

The concept of “double-aging communities” does not rely on a strict chronological definition of older adults but rather refers to neighborhoods characterized by an overall aged population structure and a high proportion of retired or semi-retired residents. Although conventional definitions often classify individuals aged 60 and above as older adults, this study included residents aged 55 and above. This age group largely represents early retirees who are already active participants in the market for older adults. Including them allows for capturing early-stage aging patterns in community distribution, activity preferences, and service needs, providing a more comprehensive understanding of aging dynamics. Moreover, extending the lower age threshold ensures sufficient sample size for robust statistical analyses and offers valuable insights into the gradual effects of aging on community environment utilization, green space accessibility, and social networks, thereby enhancing the policy relevance of the findings.

### 2.3 Questionnaire design

Based on the research objectives, this study integrated variable indicators from established domestic and international studies to design the questionnaire (51–55) (Table 1). The questionnaire items were derived from well-validated scales covering key constructs relevant to older residents’ mental health and green space use, including social interaction, planning features, overall perception of the community environment, green space activity behaviors, and psychological health.

**Table 1:** Survey constructs and measurement items for SEM.

Category	Dimension	Measurement content
Individual characteristics of older adults	Demographic characteristics	Gender (Q1), Age (Q2), Length of residence in the community (Q3), Per capita household monthly income (Q4), Education level (Q5), Home-based care pattern (Q6)
	Self-reported health status	Current health status (Q7), Satisfaction with health status (Q8)
Social features of green space environment	Social interaction frequency	Frequency of neighborly communication in green space (Q9), Mutual care in green space (Q10), Assistance in green space (Q11)
	Social recognition	Trustworthiness of people in green space (Q12), Harmonious coexistence among residents (Q13)
Planning features of green space environment	Diversity	Diversity of plant spatial layout (Q14), Diversity of landscape design types (Q15), Diversity of plant species (Q16)
	Accessibility	Convenience of walking to green spaces (Q17), Completeness of barrier-free facilities (Q18), Reasonable sidewalk planning (Q19)
	Safety	Night-time lighting (Q20), Separation of pedestrian and vehicle flows (Q21), Orderly parking management (Q22)
	Comfort	Physical environmental comfort (Q23), Suitable temperature perception (Q24), Absence of noise (Q25), Absence of allergens or unpleasant odors (Q26)
	Usability	Variety of facilities (Q27), Quantity of facilities (Q28), Convenience of use (Q29)
	Aesthetics	Aesthetic quality of landscape (Q30), Tidiness and cleanliness of environment (Q31)
Overall perception	Satisfaction	Overall satisfaction with community green space (Q32), Satisfaction with landscape aesthetics (Q33)
Green space activity behavior	Activity characteristics	Activity frequency (Q34), Activity duration (Q35), Main types of activities (Q36)
Psychological health of older adults	Positive emotions	Feeling cheerful (Q37), Energetic (Q38), Calm and relaxed (Q39), Interest in daily life (Q40), Sense of life meaning (Q41)
	Negative emotions	Frequent feelings of loneliness (Q42), Lack of companionship (Q43), Social disconnection (Q44)
	Emotional and attention restoration	Ability of natural environment to stabilize emotions (Q45), Ability to restore attention in green space (Q46)

**Figure 3:**
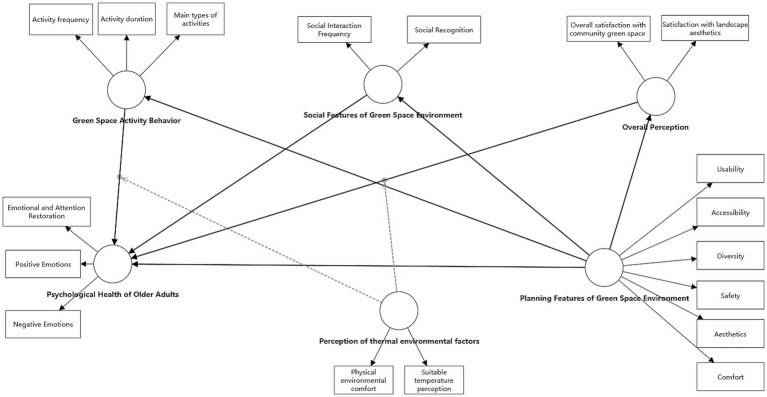
SEM-based conceptual model of factors influencing older adults mental health.

Initially, a pre-test was conducted to evaluate the measurement instrument within the specific context of local urban communities. During this stage, considering the older participants’ attention span and response capacity, the questionnaire was systematically streamlined by identifying and removing repetitive questions to reduce respondent burden. The empirical feedback from this pre-test directly informed the determination of the preliminary SEM framework (51, 55). In the subsequent formal SEM analysis, additional items were removed based on statistical criteria and model fit. These step-by-step adjustments were implemented to preserve the validity and reliability of the original constructs while ensuring the measurement instrument remained feasible. Ultimately, the finalized SEM approach was applied to analyze the complex relationships among these variables, testing both direct and indirect effects within the established causal framework (Figure 3).

### 2.4 Data analysis

#### 2.4.1 Structural equation modeling

SEM was employed to systematically examine complex causal relationships among observed and latent variables. A preliminary causal framework was first established based on theoretical reasoning and previous literature, aiming to evaluate both direct and indirect effects among latent constructs and to assess the overall model fit. The analysis followed a two-stage approach. In the first stage, the measurement model was assessed to ensure reliability, validity, and absence of multicollinearity. Internal consistency of each construct was evaluated using Cronbach’s *α* and Composite Reliability [CR ≥ 0.70; (56)], while convergent validity was examined through Outer Loadings and Average Variance Extracted [AVE ≥ 0.50; (57)]. Discriminant validity was assessed using Cross-Loadings and the Heterotrait-Monotrait ratio (HTMT < 0.90 for acceptable discriminant validity; <0.85 for strict standard; (58)). Variance inflation factors (VIFs) were checked to rule out severe multicollinearity among indicators, with all VIF values below the threshold of 5 (59).

In the second stage, the structural model was evaluated by estimating path coefficients to test hypothesized relationships between latent constructs. Bootstrapping procedures were applied to assess the statistical significance of direct and indirect effects. Model fit was comprehensively assessed using multiple indices, including explained variance [R^2^, with thresholds interpreted as small (≈0.02), medium (≈0.13), and large (≥0.26) effects]; (71), Goodness-of-Fit (GoF > 0.36 for large effect size; (72), and other relevant fit indices to confirm that the model adequately represented the data. This systematic approach ensured that the measurement properties of the constructs and the structural relationships among variables were rigorously validated, providing a robust foundation for interpreting causal relationships in the study.

#### 2.4.2 Hierarchical regression analysis

Hierarchical regression analysis was employed to examine the incremental contributions of different groups of predictors to the psychological health of older adults, which was set as the dependent variable. Predictors were entered in sequential blocks based on theoretical rationale, and the change in explained variance (ΔR^2^) was examined at each step to evaluate the additional explanatory power of newly added variables. Multicollinearity among predictors was assessed using VIF, with all values maintained below the common threshold of 5 to ensure stability of regression coefficients. Standardized regression coefficients (*β*), t-statistics, and significance levels (*p* < 0.05) were reported for each predictor. Model fit was evaluated using adjusted R^2^, with thresholds interpreted as small (≈0.02), medium (≈0.13), and large (≥0.26) effects. This approach enabled the identification of the relative importance of demographic, environmental, and social variables in influencing psychological health, and facilitated a systematic understanding of how different sets of factors jointly affect outcomes in older populations.

#### 2.4.3 Differences analysis

To examine significant differences in the data across groups, normality tests were first conducted for all variables. If the normality assumption was satisfied, one-way analysis of variance (ANOVA) or independent-sample *t*-tests were applied to evaluate differences among multiple or two groups, respectively. When the normality assumption was violated, non-parametric alternatives such as the Kruskal–Wallis test or Mann–Whitney U test were employed. Statistical significance was determined using a threshold of *p* < 0.05. This approach ensured that the choice of statistical method was appropriate for the data distribution while allowing robust identification of differences across community types or participant groups.

Data analysis for this study was conducted using SmartPLS 4, SPSS 24, and Origin 2024b.

## 3 Results

### 3.1 Descriptive statistics

To ensure balanced representation across community types, the field survey was conducted as part of a course involving four classes, each with 18 class members. Each student was assigned to collect five questionnaires from a designated community type: low building age & low population aging (LL), low building age & high population aging (LH), high building age & low population aging (HL), or high building age & high population aging (HH). After excluding invalid responses and ensuring even sampling, a total of 78 questionnaires were collected for each community type, resulting in an overall sample size of 312.

The sample was nearly balanced in terms of gender, with 150 males (48.1%) and 162 females (51.9%). The majority of participants were aged 65–74 years (42.9%), followed by 55–64 years (31.1%) and 75 years and above (26.0%). Most participants (87.5%) resided in communities that had not undergone recent improvement, while 12.5% lived in improved communities. The length of residence varied, with 17.3% residing for less than 2 years, 20.8% for 3–5 years, 19.9% for 6–10 years, 21.2% for 11–15 years, and 20.8% for 16–20 years.

Household income per capita was relatively balanced across categories: 35.6% of participants reported less than 3,000 CNY, 29.8% reported 3,000–5,000 CNY, and 34.6% reported 5,000 CNY or above. In terms of education, 28.2% of participants had completed primary school or below, 25.6% junior high school, 21.8% senior high school or technical secondary school, and 24.4% held a college degree or higher. Regarding home-based care patterns, 34.3% of participants lived alone, 34.0% lived with a spouse, and 31.7% lived with children or in a three-generation household (Table 2).

**Table 2:** Demographic characteristics of the study sample (*n* = 312).

Sample attributes	Category	Frequency	Percentage (%)
Gender	Male	150	48.1
	Female	162	51.9
Age	55–64 years	97	31.1
	65–74 years	134	42.9
	75 years and above	81	26.0
Community type	LL	78	25
	LH	78	25
	HL	78	25
	HH	78	25
Length of residence	Less than 2 years	54	17.3
	3–5 years	65	20.8
	6–10 years	62	19.9
	11–15 years	66	21.2
	16–20 years	65	20.8
Monthly household income per capita	<3,000 CNY	111	35.6
	3,000–5,000 CNY	93	29.8
	≥ 5,000 CNY	108	34.6
Education level	Primary school or below	88	28.2
	Junior high school	80	25.6
	Senior high school/technical secondary school	68	21.8
	College/bachelor or above	76	24.4
Home-based care pattern	Living alone	107	34.3
	Living with spouse	106	34.0
	Living with children or three generations	99	31.7

### 3.2 SEM measurement model assessment

#### 3.2.1 Reliability and convergent validity assessment

The reliability and convergent validity of the measurement model were assessed using Cronbach’s alpha, composite reliability (CR), and average variance extracted (AVE). As shown in Table 3, all constructs exhibited satisfactory internal consistency, with Cronbach’s alpha values ranging from 0.804 to 0.972, exceeding the recommended threshold of 0.70. Composite reliability values (both rho_a and rho_c) were all above 0.80, indicating strong construct reliability. In addition, all AVE values exceeded the recommended level of 0.50, ranging from 0.758 to 0.967, confirming adequate convergent validity (56, 57).

**Table 3:** Reliability and convergent validity of latent constructs.

Latent construct	Cronbach’s α	Composite reliability (rho_a)	Composite reliability (rho_c)	Average variance extracted (AVE)
Overall perception	0.804	0.815	0.910	0.835
Thermal environment	0.824	0.853	0.918	0.849
Social features	0.966	0.967	0.983	0.967
Green space activity types	0.841	0.849	0.904	0.758
Psychological health of older adults	0.972	0.972	0.982	0.947
Planning features	0.964	0.966	0.971	0.849

#### 3.2.2 Indicator loadings and discriminant validity assessment

To further evaluate the quality of the measurement model, indicator loadings and discriminant validity were examined. As presented in Table 4, all outer loadings exceeded the recommended threshold of 0.60, indicating that each indicator makes a substantial contribution to its corresponding latent construct (56).

**Table 4:** Outer loadings of indicators for latent constructs.

**Indicator**	**Thermal Environment**	**Overall Perception**	**Social Features**	**Green Space Activity Types**	**Planning Features**	**Psychological Health of Older Adults**
Physical environmental comfort	0.903					
Suitable temperature perception	0.939					
Overall satisfaction with community green space		0.901				
Satisfaction with landscape aesthetics		0.926				
Social Interaction Frequency			0.983			
Social Recognition			0.984			
Activity frequency				0.881		
Activity duration				0.846		
Main types of activities				0.884		
Usability					0.957	
Accessibility					0.860	
Diversity					0.902	
Safety					0.940	
Aesthetics					0.910	
Comfort					0.954	
Emotional and Attention Restoration						0.970
Positive Affect						0.972
Negative Affect						0.976

Discriminant validity was assessed using three complementary approaches: cross-loadings, the Fornell–Larcker criterion, and the Heterotrait–Monotrait ratio (HTMT). First, the cross-loading results (Table 5) show that each indicator loads more strongly on its associated construct than on any other construct, confirming adequate discriminant validity. Second, the Fornell–Larcker criterion (Table 6) indicates that the square roots of the AVE values on the diagonal are higher than the inter-construct correlations, further supporting discriminant validity. Finally, the HTMT results (Table 7) reveal that all values are below the conservative threshold of 0.85, providing additional evidence of satisfactory discriminant validity (58).

**Table 5:** Cross-loadings of indicators.

**Indicator**	**Overall Perception**	**Thermal Environment Factors**	**Social Characteristics**	**Types of Green Space Activities**	**Mental health level of older adults**	**Planning Features**
Physical environmental comfort	0.216	0.903	0.193	0.230	0.198	0.237
Suitable temperature perception	0.197	0.939	0.149	0.248	0.247	0.251
Overall satisfaction with community green space	0.901	0.221	0.338	0.394	0.340	0.334
Satisfaction with landscape aesthetics	0.926	0.188	0.323	0.433	0.412	0.363
Social Interaction Frequency	0.401	0.239	0.284	0.881	0.374	0.304
Social Recognition	0.384	0.187	0.293	0.846	0.296	0.257
Social Interaction Frequency	0.399	0.248	0.296	0.884	0.346	0.293
Interaction Frequency	0.348	0.184	0.983	0.324	0.393	0.435
Accessibility	0.345	0.250	0.407	0.298	0.386	0.957
Reachability	0.330	0.235	0.377	0.315	0.402	0.860
Diversity	0.396	0.226	0.504	0.297	0.424	0.902
Safety	0.382	0.244	0.404	0.307	0.421	0.940
Emotion and Attention Restoration	0.402	0.234	0.411	0.373	0.970	0.430
Social Environment Recognition	0.361	0.175	0.984	0.332	0.399	0.448
Positive Emotion	0.400	0.241	0.377	0.398	0.972	0.412
Aesthetic Quality	0.301	0.249	0.366	0.282	0.405	0.910
Comfort	0.348	0.264	0.408	0.317	0.380	0.954
Negative Emotion	0.406	0.237	0.388	0.372	0.976	0.438

**Table 6:** Fornell-Larcker criterion for discriminant validity.

Latent construct	Overall perception	Thermal environment	Social features	Green space activity types	Psychological health of older adults	Planning features
Overall perception	0.914					
Thermal environment	0.222	0.921				
Social features	0.361	0.183	0.984			
Green space activity types	0.454	0.260	0.334	0.871		
Psychological health of older adults	0.414	0.244	0.403	0.392	0.973	
Planning features	0.382	0.265	0.449	0.329	0.439	0.921

**Table 7:** Discriminant validity assessment: Heterotrait-Monotrait ratio (HTMT).

Latent construct	Overall perception	Thermal environment	Social features	Green space activity types	Psychological health of older adults	Planning features
Overall perception						
Thermal environment	0.277					
Social features	0.410	0.208				
Green space activity types	0.550	0.309	0.371			
Psychological health of older adults	0.465	0.269	0.416	0.430		
Planning features	0.431	0.297	0.462	0.363	0.452	

Collectively, these assessments confirm that the measurement model exhibits strong reliability, convergent validity, and discriminant validity, supporting its suitability for subsequent structural model analysis.

#### 3.2.3 Multicollinearity assessment

Multicollinearity among constructs was assessed using the variance inflation factor (VIF). As shown in Table 8, all VIF values are below the conservative threshold of 5.0, indicating that multicollinearity is not a concern in the structural model. This confirms that the predictors are sufficiently independent for subsequent structural analysis.

**Table 8:** Variance inflation factor (VIF) assessment of latent constructs.

Latent construct	Overall perception	Thermal environment	Social features	Green space activity types	Psychological health of older adults	Planning features
Overall perception						1.569
Thermal environment						1.128
Social features						1.355
Green space activity types						1.528
Psychological health of older adults						
Planning features	1.000		1.000	1.000	1.445	

#### 3.2.4 Overall model fit assessment

To evaluate the overall fit of the structural equation model, the Goodness-of-Fit (GoF) index was computed using the geometric mean of the Average Variance Extracted (AVE) and the average R^2^ value of the endogenous constructs. The GoF formula is defined as follows:


$$\mathrm{GoF}=\sqrt{\bar{\mathrm{AVE}}\times {\bar{R}}^{2}}$$

Based on the measurement and structural model assessments, the average AVE ( $\bar{\mathrm{AVE}}$ ) across all latent variables was 0.8675 , and the average R^2^ ( ${\bar{R}}^{2}$ ) for the endogenous variables was 0.2148 . Substituting these values into the formula yields:


$$\mathrm{GoF}=\sqrt{0.8675\times 0.2148}=\sqrt{0.1863}\approx 0.432$$

The calculated GoF value of 0.432 exceeds the established threshold for a large effect size (GoF > 0.36), indicating that the model provides an adequate representation of the relationships among constructs. This result supports the robustness of the model and provides a reliable framework for interpreting the influence of environmental and social factors on older adults’ psychological health.

### 3.3 Structural equation model results

**Figure 4:**
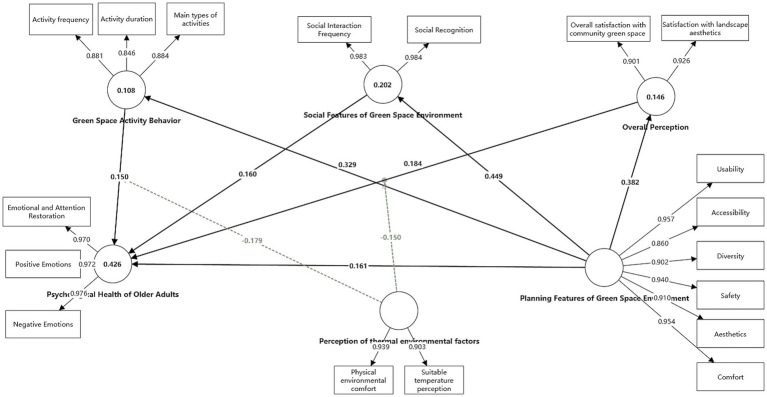
SEM results depicting pathways through which various factors influence older adults’ psychological health.

Overall, the model demonstrates a well-structured pathway linking green space planning, environmental perception, behavioral engagement, and psychological health outcomes among older adults. Planning features exert both direct and indirect effects on psychological health through multiple mediating pathways, including overall perception, social features, and green space activity behavior. In addition, significant moderating effects of thermal environmental factors were observed. Specifically, the interaction terms between thermal environment and both overall perception and green space activity behavior showed significant negative effects on psychological health, indicating that high thermal stress conditions weaken the positive effects of environmental perception and activity engagement (Figure 4).

#### 3.3.1 Estimation of path coefficients

The results of path coefficient estimation are presented in Table 9. Direct effects indicate that overall perception (*β* = 0.184, *p* = 0.001), social features (*β* = 0.160, *p* = 0.007) and green space activity behavior (*β* = 0.150, *p* = 0.008) all have significant positive effects on the psychological health of older adults. Among the antecedent variables, planning features show the strongest explanatory power, exerting significant positive effects on overall perception (*β* = 0.382, *p* < 0.001), social features (*β* = 0.449, *p* < 0.001), and green space activity behavior (*β* = 0.329, *p* < 0.001), as well as a direct effect on psychological health (*β* = 0.161, *p* = 0.010). This suggests that planning-level interventions play a central role in shaping both environmental perception and behavioral engagement. Regarding moderating effects, thermal environment × overall perception (*β* = −0.150, *p* = 0.003) and thermal environment × green space activity behavior (*β* = −0.179, *p* < 0.001) both exhibit significant negative effects on psychological health, indicating that thermal stress conditions attenuate the beneficial effects of environmental cognition and behavioral participation.

**Table 9:** Path coefficients of the structural equation model.

Structural Paths	Original sample (O)	Standard deviation (STDEV)	T statistics (|O/STDEV|)	*p*-values
Overall Perception → Psychological Health of Older Adults	0.184	0.058	3.189	0.001
Thermal Environment → Psychological Health of Older Adults	0.093	0.047	1.989	0.047
Social Features → Psychological Health of Older Adults	0.16	0.06	2.693	0.007
Green Space Activity Types → Psychological Health of Older Adults	0.15	0.056	2.664	0.008
Planning Features → Overall Perception	0.382	0.06	6.33	0
Planning Features → Social Features	0.449	0.058	7.713	0
Planning Features → Green Space Activity Types	0.329	0.064	5.151	0
Planning Features → Psychological Health of Older Adults	0.161	0.063	2.564	0.01
Thermal Environment × Overall Perception → Psychological Health of Older Adults	-0.15	0.051	2.933	0.003
Thermal Environment × Green Space Activity Types → Psychological Health of Older Adults	-0.179	0.049	3.62	0

#### 3.3.2 Model explanatory and predictive power

The model demonstrates satisfactory explanatory power for all endogenous constructs. The R^2^ values are 0.146 (overall perception), 0.202 (social features), 0.108 (green space activity behavior), and 0.426 (psychological health), indicating moderate explanatory power for behavioral and social constructs and relatively strong explanatory power for psychological health outcomes.

The adjusted R^2^ values show consistent results, confirming model stability (Table 10). Effect size analysis (f^2^) indicates that planning features exert the strongest influence on overall perception (*f*^2^ = 0.171), social features (*f*^2^ = 0.253), and green space activity behavior (*f*^2^ = 0.121), while its direct effect on psychological health is relatively smaller (*f*^2^ = 0.031) but still meaningful (Table 11). Predictive relevance assessment (Q^2^) shows values ranging from 0.431 to 0.827, indicating strong predictive relevance (Table 12).

**Table 10:** R^2^ and adjusted *R*^2^-values of latent variables.

Latent variable	*R* ^2^	Adjusted *R*^2^
Overall perception	0.146	0.143
Social features	0.202	0.199
Green space activity types	0.108	0.105
Psychological health of older adults	0.426	0.412

**Table 11:** f^2^ effect sizes of independent variables on dependent variables.

Independent variable	Overall perception	Thermal environment	Social features	Green space activity types	Psychological health of older adults	Planning features
Overall perception						0.038
Thermal environment						0.013
Social features						0.033
Green space activity types						0.026
Psychological health of older adults						
Planning features	0.171		0.253	0.121	0.031	

*f*^2^-values indicate the effect size of each independent latent variable on a dependent latent variable. According to Cohen (71), *f*^2^-values of 0.02, 0.15, and 0.35 correspond to small, medium, and large effect sizes, respectively.

**Table 12:** Q^2^ predictive relevance of latent variables.

Latent variable	SSO	SSE	Q^2^ (= 1 – SSE/SSO)
Overall perception	624	354.876	0.431
Thermal environment	624	335.796	0.462
Social features	624	176.181	0.718
Green space activity types	936	476.071	0.491
Psychological health of older adults	936	161.870	0.827
Planning features	1872	409.945	0.781

*Q*^2^-values indicate the predictive relevance of each latent variable. A *Q*^2^-value greater than 0 suggests that the model has predictive relevance for the corresponding construct (56).

#### 3.3.3 Hierarchical regression results

To examine the incremental explanatory power of different groups of variables, hierarchical regression analysis was conducted with psychological health of older adults as the dependent variable. Six models were estimated sequentially (*n* = 312), and the results are presented in Table 13.

**Table 13:** Hierarchical regression results (*n* = 312).

Observed variable	Model 1	Model 2	Model 3	Model 4	Model 5	Model 6
	Standardized β	Standardized β	Standardized β	Standardized β	Standardized β	Standardized β
Gender	0.007	0.015	0.053	0.054	0.079	0.095
Age	0.031	0.000	−0.018	−0.025	0.001	−0.002
Community type	0.141	0.104	0.089	0.081	0.041	0.031
Community improvement	0.172	0.124	0.101	0.093	0.068	0.061
Length of residence	0.081	0.037	0.044	0.035	0.020	0.022
Monthly household income per capita	−0.003	0.038	0.030	0.036	0.054	0.048
Education level	−0.018	−0.029	−0.021	−0.017	−0.029	−0.041
Home-based care pattern		0.077	0.049	0.047	0.033	0.016
Social interaction frequency		0.378	0.250	0.106	0.080	0.076
Diversity			0.025	−0.224	−0.252	−0.160
Accessibility			0.132	−0.103	−0.084	−0.070
Safety			0.230	−0.002	−0.020	0.021
Comfort			−0.288	−0.467	−0.459	−0.557
Usability			0.060	−0.142	−0.132	−0.002
Aesthetics			0.204	0.059	0.110	0.124
Green space planning features				1.201	1.087	0.880
Physical environmental comfort				0.053	0.037	0.023
Satisfaction with residential green space					0.024	0.009
Satisfaction with green space aesthetics					0.243	0.195
Activity frequency						0.175
Activity duration						−0.026
Primary activity type						0.049
*R* ^2^	0.034	0.177	0.278	0.284	0.335	0.362
Adjusted *R*^2^	0.011	0.153	0.242	0.242	0.292	0.314
*F*-value	*F*(7, 304) = 1.510, *p* = 0.163	*F*(9, 302) = 7.237, *p* < 0.001	*F*(15, 296) = 7.607, *p* < 0.001	*F*(17, 294) = 6.846, *p* < 0.001	*F*(19, 292) = 7.735, *p* < 0.001	*F*(22, 289) = 7.462, *p* < 0.001

Dependent variable = psychological health of older adults. *p* < 0.05, **p < 0.01, ***p < 0.001.

Model 1, which included only demographic variables, showed limited explanatory power (*R*^2^ = 0.034, adjusted *R*^2^ = 0.011) and was not statistically significant (*p* = 0.163), indicating weak direct associations between demographic characteristics and psychological health.

After introducing social features in Model 2, the explanatory power increased significantly (*R*^2^ = 0.177, Δ*R*^2^ = 0.143, *p* < 0.001), with frequency of social interaction showing a significant positive effect (Tables 14–16).

**Table 14:** Differences in overall perception and psychological health before and after community renovation.

Variable	Non-renovated (*n* = 39) Mean ± SD	Renovated (*n* = 39) Mean ± SD	*t*	*p*
Overall perception	2.94 ± 1.17	3.58 ± 1.07	−2.520	0.014*
Psychological health of older adults	2.98 ± 0.99	3.60 ± 0.90	−2.892	0.005**

Independent-samples *t*-tests were conducted to compare the means before and after community renovation. *p < 0.05, **p < 0.01, ***p < 0.001.

**Table 15:** Differences in overall perception and psychological health before and after community renovation among older adults aged 55–65.

Variable	Non-renovated (*n* = 25) Mean ± SD	Renovated (*n* = 24) Mean ± SD	*t*	*p*
Overall perception	2.92 ± 1.19	3.81 ± 1.06	−2.770	0.008**
Psychological health of older adults	2.83 ± 0.99	3.63 ± 0.99	−2.820	0.007**

Independent-samples *t*-tests were conducted to examine the effects of community renovation on middle-aged older adults (55–65 years). *p < 0.05, **p < 0.01, ***p < 0.001.

**Table 16:** Differences in overall perception and psychological health before and after community renovation among older adults aged 65 and above.

Variable	Non-renovated (*n* = 7) Mean ± SD	Renovated (*n* = 5) Mean ± SD	*t*	*p*
Overall perception	2.93 ± 1.06	2.70 ± 0.84	0.400	0.697
Psychological health of older adults	3.53 ± 0.93	3.74 ± 0.78	−0.406	0.694

Independent-samples *t*-tests were conducted to examine the effects of community renovation on older adults aged 65 and above. *p < 0.05, **p < 0.01, ***p < 0.001.

In Model 3, green space planning variables were added, leading to further improvement in model fit (*R*^2^ = 0.278, Δ*R*^2^ = 0.101, *p* < 0.001). However, most planning indicators did not show statistically significant effects, and their coefficients varied in magnitude and direction.

Model 4 introduced environmental and thermal-related factors, resulting in only a marginal improvement in explanatory power (*R*^2^ = 0.284, Δ*R*^2^ = 0.006), indicating limited additional explanatory contribution.

In Model 5, perceptual variables were included, further increasing explanatory power (*R*^2^ = 0.335, Δ*R*^2^ = 0.051, *p* < 0.001). Among them, satisfaction with green space aesthetics showed a significant positive effect, while general satisfaction was not significant.

Finally, Model 6 incorporated behavioral variables, resulting in the highest explanatory power (*R*^2^ = 0.362, Δ*R*^2^ = 0.027, *p* < 0.001), with activity frequency showing a significant positive effect, whereas activity duration and activity type remained non-significant.

Overall, the hierarchical regression results suggest that social interaction, perceptual evaluation, and activity frequency are the most consistent predictors of psychological health, whereas demographic characteristics and most planning-related variables show limited direct effects.

**Figure 5:**
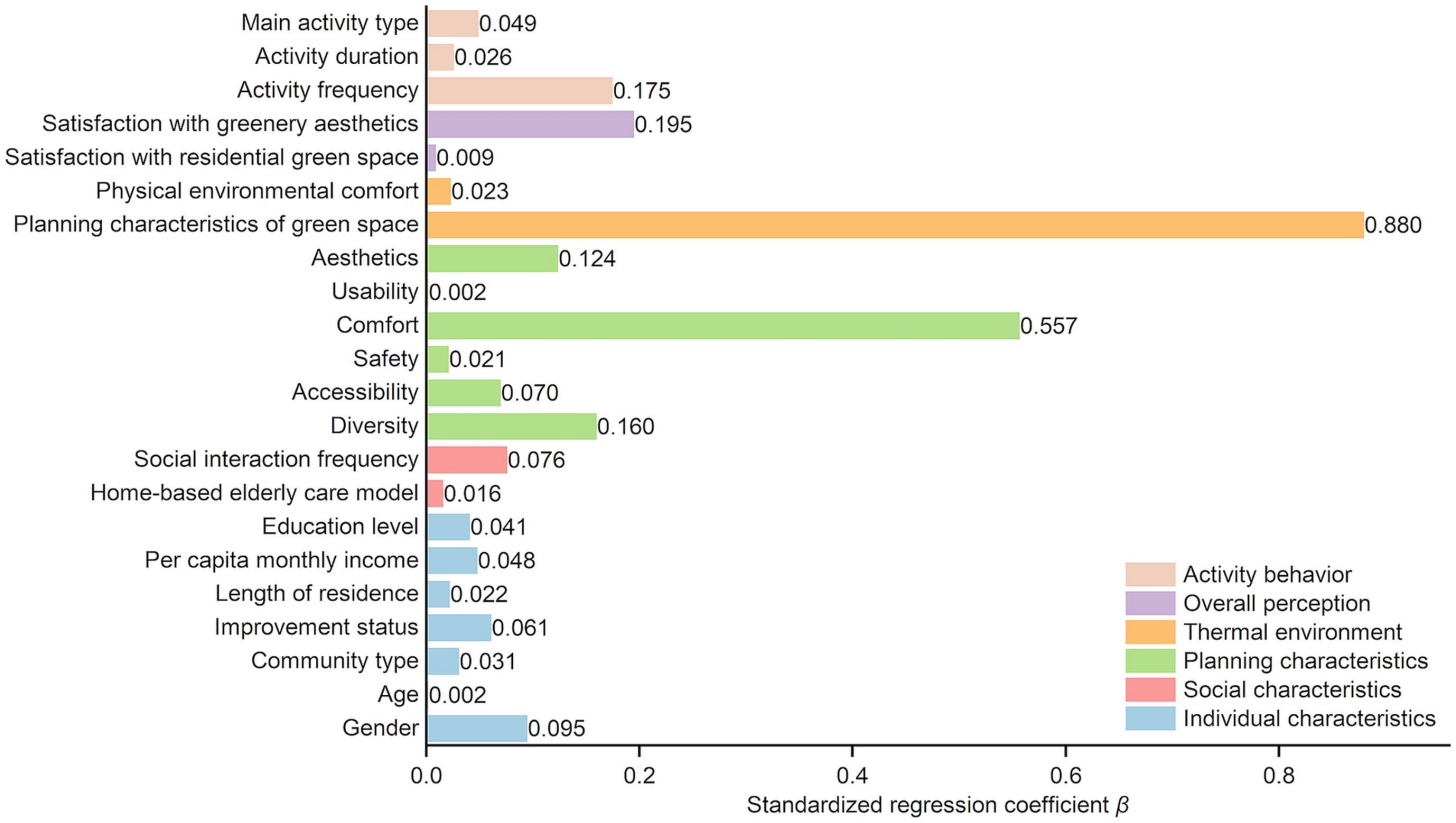
Standardized regression coefficients (*β*) of individual predictors on older adults psychological health.

To further examine the relative importance of individual predictors, standardized regression coefficients (*β*) from the final model were analyzed. The results indicate substantial heterogeneity across variables. Within planning-related indicators, comfort (*β* = 0.557) and overall environmental quality (*β* = 0.880) exhibit relatively stronger effects, while accessibility, safety, and usability show negligible contributions. In contrast, behavioral and perceptual variables, including activity frequency (*β* = 0.175) and aesthetic satisfaction (*β* = 0.195), demonstrate moderate but stable positive effects. Individual and social characteristics generally exhibit weak effects (*β* < 0.10), indicating limited direct explanatory power in the regression framework. Overall, psychological health appears to be more strongly associated with behavioral engagement and perceptual experience than with demographic attributes or most planning-related indicators (Figure 5).

### 3.4 Differences analysis

#### 3.4.1 Community renovation effects

**Figure 6:**
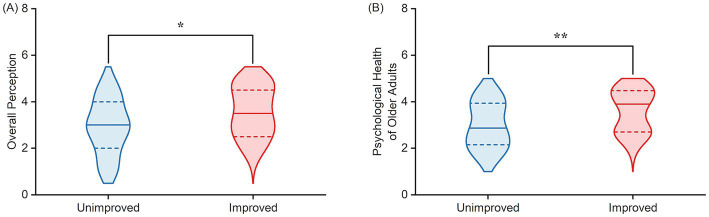
Impact of community renovation on perception and psychological health of older adults. **(A)** Overall perception; **(B)** psychological health of older adults. *p < 0.05; **p < 0.01.

To evaluate the impact of community renovation on older adults’ overall perception and psychological health, independent-samples *t*-tests were conducted between residents in renovated and non-renovated old-building communities (*n* = 78). The results demonstrated that community renovation significantly improved both overall perception and psychological health (overall perception: *t* = −2.520, *p* = 0.014; psychological health: *t* = −2.892, *p* = 0.005), indicating that environmental improvements can effectively enhance residents’ subjective evaluation of the community and their psychological wellbeing (Figure 6, Table 14).

#### 3.4.2 Age-specific effects

**Figure 7:**
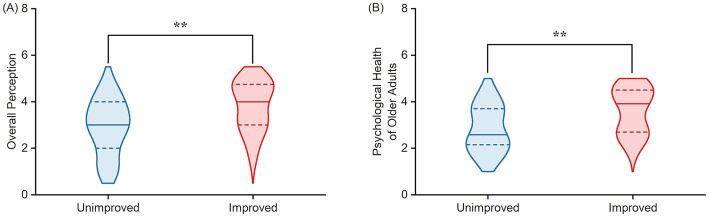
Impact of community renovation on perception and psychological health among adults aged 55–65 years. **(A)** Overall perception. **(B)** Psychological health of adults aged 55–65 years. **p < 0.01.

Further analysis revealed age-specific differences in response to community renovation. Among middle-aged older adults (55–65 years), residents in renovated communities reported significantly higher overall perception and psychological health compared to those in non-renovated communities (overall perception: *t* = −2.770, *p* = 0.008; psychological health: *t* = −2.820, *p* = 0.007), suggesting that renovation has a notable positive impact on this age group (Figure 7, Table 15).

**Figure 8:**
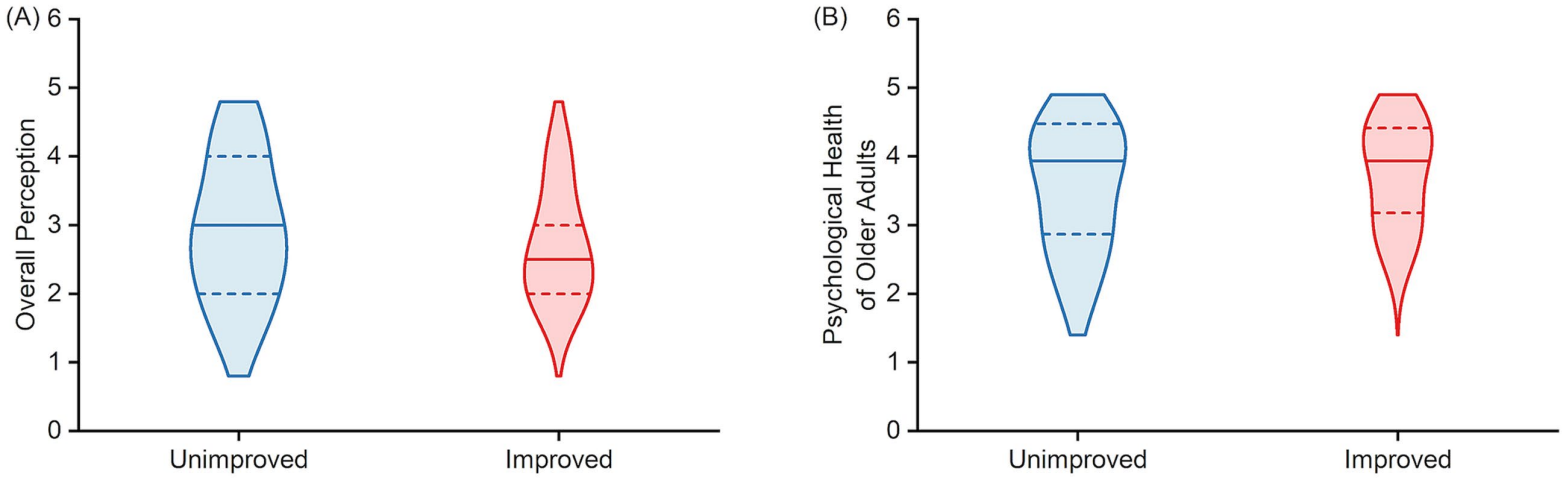
Impact of community renovation on perception and psychological health among older adults aged 65 years and above. **(A)** Overall perception. **(B)** Psychological health.

In contrast, older adults aged 65 + and above did not exhibit significant differences in either overall perception or psychological health (overall perception: *t* = 0.400, *p* = 0.697; psychological health: *t* = −0.406, *p* = 0.694), likely due to physical limitations, reduced mobility, or other individual constraints. Overall, the findings indicate that community renovation has a more pronounced effect on middle-aged older adults, while its impact on the oldest cohort is limited (Figure 8, Table 16).

## 4 Discussion

### 4.1 Mechanism of green space effects on psychological health

Based on SEM, hierarchical regression, and group comparison analyses, this study reveals a multi-level and pathway-dependent mechanism through which green space environments influence the psychological health of older adults in “dual-aging” communities. The SEM results indicate that green space planning features exert limited direct effects on psychological health. Instead, their primary function lies in shaping residents’ environmental perceptions, opportunities for social interaction, and engagement in green space activities, which in turn significantly affect psychological health outcomes. This finding suggests that the influence of green spaces is largely indirect and mediated, rather than directly structural, which is consistent with prior research indicating that social interactions, perceived environmental quality, and physical activity are key mediators between green spaces and mental wellbeing (60–63). Hierarchical regression analysis further supports this mechanism, showing that social interaction frequency, aesthetic satisfaction with green space, and activity frequency are the most consistent predictors of psychological health, whereas most demographic variables and individual planning indicators do not reach statistical significance. These results highlight that the psychological benefits of urban green spaces for older adults primarily depend on how these spaces are perceived and used, rather than on physical configuration alone.

### 4.2 Heterogeneity of planning attributes and the weak role of accessibility

A key finding of this study is the substantial heterogeneity among planning-related variables. While attributes such as environmental comfort and overall quality show relatively stronger effects, other indicators—including accessibility, safety, and usability—exhibit weak or non-significant effects. In particular, the weak effect of accessibility appears to differ from previous studies, in which accessibility has often been identified as a core determinant of the benefits of green spaces (32, 36). This discrepancy may be attributed to the specific spatial context of this study. At the intra-community scale within a dense urban core, most residential units are already located within walking distance of green spaces, resulting in limited variability in accessibility and a potential ceiling effect. Moreover, for older adults, especially those living in long-established communities, daily activity ranges tend to be highly localized and stable. In this context, accessibility functions primarily as a basic prerequisite rather than a differentiating factor, whereas variations in environmental quality and experiential attributes (e.g., comfort and aesthetics) become more influential in shaping psychological outcomes. These findings suggest that the importance of accessibility is context-dependent and may diminish in environments where baseline spatial access is already satisfied.

### 4.3 Age-specific response mechanism

The results also indicate clear age-based heterogeneity in the effects of environmental improvement. The 55–65 age group shows significantly stronger responses to green space improvement compared with older cohorts (65 + years). This phenomenon may be partly explained by the transitional life stage of this group in the Chinese context. Many individuals in this age range are in a early retirement stage or early retirement stage, characterized by increased discretionary time and reduced work-related social interactions. At the same time, they generally maintain relatively good physical mobility and social engagement capacity. As a result, they may exhibit stronger behavioral responsiveness to environmental improvements, with community green spaces serving as important venues for social interaction and daily activity. In contrast, older cohorts may experience physical limitations and reduced mobility, constraining their ability to fully benefit from environmental enhancements (64).

### 4.4 Moderating role of thermal environment

The SEM results further show that thermal environmental conditions significantly weaken the positive effects of environmental perception and green space activity on psychological health. This indicates that the benefits of green space use are highly dependent on microclimatic comfort conditions. High-temperature conditions may reduce outdoor activity participation and weaken the restorative experience of green spaces. This finding aligns with previous studies emphasizing the importance of climatic comfort in shaping urban outdoor behavior (65, 66). It suggests that green space planning should not only focus on spatial configuration but also incorporate microclimate regulation strategies, such as shading, ventilation, water features, and cooling infrastructure.

Overall, the findings converge to an integrated mechanism in which green space planning functions as an upstream structural driver that influences psychological health indirectly through environmental perception and behavioral engagement, while thermal conditions and age act as important boundary conditions shaping the strength of these relationships. Specifically, green space planning first affects residents’ subjective evaluations of the environment, which then influences their frequency and type of green space use. These behavioral processes subsequently contribute to psychological health outcomes. However, this pathway is moderated by climatic conditions and individual capacity, and is not uniformly distributed across different population groups. This integrated perspective helps reconcile differences between SEM and regression results and highlights that the health benefits of urban green space emerge from a complex interaction between spatial configuration, subjective perception, behavioral participation, and environmental constraints, rather than from physical design alone.

## 5 Conclusion

### 5.1 Theoretical and practical implications

From a theoretical perspective, this study extends existing environmental health research by emphasizing the mediated and context-dependent nature of green space effects on psychological health. It highlights the importance of distinguishing between perception-level and outcome-level variables, as well as recognizing the temporal lag between environmental improvement and health outcomes.

From a practical perspective, the findings suggest that improving psychological health in “double-aging” communities requires more than increasing green space quantity or accessibility. Priority should be given to enhancing environmental comfort, aesthetic quality, and activity-supportive design, while also considering microclimatic conditions and age-specific needs. In particular, interventions targeting high-frequency social interaction and daily use behaviors may be more effective than purely spatial redesign strategies.

### 5.2 Limitations and future research

Despite its contributions, this study has several limitations. First, the sample is limited to communities in Hangzhou’s Shangcheng District, which may limit generalizability. Second, the cross-sectional design restricts causal inference. Third, psychological health and environmental perceptions are based on self-reported data, which may introduce subjective bias. Fourth, other environmental factors such as noise, air quality, and traffic safety were not fully considered. Finally, the assessment of the thermal environment relied solely on residents’ subjective perceptions of comfort, without validation against objective microclimate or meteorological data.

Overall, this study contributes to existing literature by providing an integrated understanding of the environment–perception–behavior–health pathway in urban aging contexts, and by emphasizing the context-dependent and heterogeneous nature of green space effects on psychological wellbeing. Future research could expand to multi-city comparisons, adopt longitudinal designs to capture dynamic effects, and incorporate multi-dimensional environmental indicators to build a more comprehensive environment–behavior–health framework.

## Data Availability

The raw data supporting the conclusions of this article will be made available by the authors, without undue reservation.
